# Conditions Simulating Enlargement of the Liver.—II

**Published:** 1909-05-22

**Authors:** 


### CONDITIONS SIMULATING ENLARGEMENT OF THE LIVER.—II.

Enlargements of the liver may be mistaken for, and require to be distinguished from—Firmly contracted recti abdominales muscles; kidney tumours; supra-renal tumours; pancreatic tumours; omental tumours; carcinoma of the stomach; carcinoma of the colon; fæcal accumulation in the colon; distension of the gall-bladder.

### Renal and Hepatic Enlargement.

It is not likely that a kidney tumour will be mistaken for a hepatic enlargement unless it is very large or unless it becomes adherent to the under surface of the liver; and a liver tumour is not often mistaken for a renal tumour unless the liver is dislocated or deformed and constricted by tight-lacing. A kidney tumour, if sufficiently large to be mistaken for an enlargement of the liver, would cause more lateral bulging of the abdominal wall unless the tumour of the liver happened to be a large hydatid cyst, an abscess, or a malignant growth arising from the outer and lower part of its right lobe. A tumour with a well-defined sharp edge is not the kidney. A renal tumour is rounded.

If an enlarged right kidney is not adherent to the under surface of the liver bimanual palpation may possibly show that the liver can be felt to move downwards over the renal tumour on deep inspiration, and independently of it, seeing that the movement of the liver with respiration is nearly always far greater than is that of the kidney. If the kidney is adherent to the under surface of the liver, both would move together at the same rate and to the same extent; but it might be possible to feel the relatively sharp, well-defined edge of the liver lying over the anterior surface of the upper part of the renal tumour, especially if palpation of the liver edge is begun in the epigastrium, and the edge traced thence towards the right.

The loin is more filled out and rendered more resistant behind in the case of a renal tumour than in that of a hepatic enlargement. If a marked sensation of nausea is produced as a result of manipulating the tumour it would point to the kidney as the organ involved. Another point which may be determined by bimanual palpation is that the hands can usually be placed between the costal margin and the upper part of a renal tumour. In cases of hepatic enlargement, on the other hand, the liver comes down close under the abdominal parietes, and the hand cannot be placed between it and the costal margin except in rare cases of extreme dislocation of the organ. If on percussion the normal resonant note obtained in the loin over the colon is replaced by dulness it would point to the kidney as the organ involved unless there were ascites, of which the rest of the abdomen would probably afford distinctive physical signs.

There is usually a resonant note over the front of a renal tumour on account of the colon being displaced forward and lying in front of it; the anterior surface of a liver enlargement on the other hand is generally dull, at any rate to light percussion. Occasionally, but very rarely, a coil of distended intestine may get between the edge of the liver and the abdominal parietes, causing even the lightest percussion to give a resonant note. On the other hand if the renal tumour is very large, and the colon is pushed forward and to the inner side, there may be dulness all over the front of the tumour; even when this is so, however, a band of resonance can usually be made out between the dulness of the liver, and that of the renal tumour.

Hæmaturia, pyuria, or albuminuria would be in favour of the tumour being renal, whereas jaundice or ascites would point to its being hepatic. It should be remembered, however, that these symptoms are not absolutely diagnostic of either condition, for jaundice may result from compression of the bile ducts by an enlarged or movable kidney, and hæmaturia may be due to renal infarction in a case of infective endocarditis with big nutmegged liver.

*Malignant disease of the right supra-renal gland* is decidedly rare, but it may give rise to a large tumour which is difficult to diagnose from an enlargement of the liver. The physical signs of a supra-renal tumour are practically the same as those of a renal tumour, except that the colon is usually pushed downwards instead of forwards. If the mass should become adherent to the liver it would move with it, and at the same rate as it, during respiration. In children the fact that an abnormal development of hair, of the genitals, and of the adipose tissues is sometimes noticed in association with supra-renal tumours may be helpful in arriving at the diagnosis.

*A Carcinoma of the colon or stomach*, if adherent to the liver, is apt to simulate a tumour of the latter. In the case of carcinoma of the stomach, which would most likely be of the pylorus, there would in all probability be gastric dilatation with the characteristic signs and symptoms—local abdominal distension, visible peristaltic waves, succussion splash, tympanitic percussion note, vomiting of large quantities of brown, frothy, thick, grumous fluid, hæmatemesis, and so forth. In the case of carcinoma of the colon, there might be signs of chronic intestinal obstruction—constipation, vomiting, tympanitic distension of the intestines, and possibly the passage of blood per rectum.

*(To be concluded.)*

